# Assessment of a bacterial quantification assay to detect and monitor *Pseudomonas aeruginosa* infection in patients with bronchiectasis

**DOI:** 10.1183/23120541.01389-2025

**Published:** 2026-08-03

**Authors:** Daniela Alferes de Lima Headley, Adam J. Bell, Rebecca C. Hull, Marie-Jeanne H.C. Kempen, Migle Young, Chandani Hennayake, Qingyou Du, Rachel Galloway, Eve McIntosh, Zsofia Eke, Hollian Richardson, Merete B. Long, Sarah Williams-Macdonald, Hazel Buchanan, Edward Mountjoy, Mateja Sborchia, Pawlina Dand, Daniel L. Halligan, James A. Long, Maia Kavanagh Williamson, Georgina Vernon, Declan Fawcett-Gibson, Daniel De Vega, Oriol Sibila, Stefano Aliberti, Charles S. Haworth, Rachel Dakin, Rebecca Holmes, Carolyn Clarke, James D. Chalmers

**Affiliations:** 1 Division of Respiratory Medicine and Gastroenterology, University of Dundee, Ninewells Hospital and Medical School, Dundee, UK; 2 LifeArc, Edinburgh, UK; 3 Pneumology Service, Hospital Clinic, IDIBAPS, CIBERES, University of Barcelona, Barcelona, Spain; 4 Department of Biomedical Sciences, Humanitas University, Milan, Italy; 5 Cambridge Centre for Lung Infection, Royal Papworth Hospital NHS Foundation Trust, Cambridge, UK; 6 University of Cambridge, Cambridge, UK; 7 Radcliffe Department of Medicine, University of Oxford, Oxford, UK

## Abstract

**Background:**

*Pseudomonas aeruginosa* (PA) is the most commonly detected pathogen in bronchiectasis. The clinical standard of care for pathogen detection is culture, which has low sensitivity. Early detection and improved monitoring could be used to enhance eradication and long-term suppressive treatments.

**Methods:**

A novel bacterial quantification assay (BQA) targeting ribosomal RNA (rRNA) for the detection of viable PA was developed. The BQA sensitivity and specificity in patient samples was assessed utilising culture (n=57), as well as BioFire pneumonia panel (n=111) and 16S rRNA gene sequencing (n=62). The potential clinical impact of the BQA in clinical settings was explored using two international patient cohorts.

**Results:**

The BQA detected PA in 100% of samples where PA was identified by other methods. The BQA quantification was positively correlated with quantitative culture (r=0.42; p<0.001) and BioFire (r=0.68; p<0.001). The BQA identified PA in an additional 23–44% of cases. To evaluate if this increased detection had clinical implications, we tested sputum samples considered to be PA negative by culture in patients who subsequently tested positive for PA. The BQA detected PA in 8 out of 20 patient samples 8–484 days prior to primary detection by culture. In patients considered to have successful PA eradication by culture, the BQA detected PA in 13 samples from 27 patients who went on to relapse.

**Conclusions:**

The BQA is a highly sensitive detection and quantification method for PA. The BQA demonstrated improved detection and treatment monitoring compared to culture, identifying patients who went on to either isolate a first PA or relapse.

## Shareable abstract

**A novel bacterial quantification assay (BQA) targeting ribosomal RNA for the detection of viable *P. aeruginosa* from clinical samples in bronchiectasis has been developed. The BQA shows promise as an early detection diagnostic and treatment monitoring tool.** https://bit.ly/4rf7rnr

## Introduction

Bronchiectasis is a chronic lung disease characterised by irreversible enlargement of the bronchi, inflammation, infection and airway dysfunction [1–3]. Patient symptoms include sputum production, fatigue, chronic cough and recurrent respiratory infections [4, 5]. Bronchiectasis was initially described as a rare disease; however, recent decades have seen an increase in diagnosis worldwide, with reported rates between 53 and 1249 per 100 000 individuals dependent on geographical location [6, 7].

A challenging aspect of disease management is recurrent and chronic respiratory infections. The most common pathogens detected in patient samples are *Pseudomonas aeruginosa*, *Haemophilus influenzae*, Enterobacteriaceae, *Staphylococcus aureus*, *Streptococcus pneumoniae* and *Moraxella catarrhalis* [4]. The prevalence of each pathogen is dependent on geographical location, with *P. aeruginosa* being the most common overall [4]. *P. aeruginosa* is of particular clinical importance as it is associated with increased exacerbation frequency, risk of hospitalisation and mortality [8, 9].

Clinical care for patients with bronchiectasis is guided exclusively by traditional bacterial culture methods [10, 11]. Culture-based methods, however, have low sensitivity and may not detect organisms present at low bacterial loads or otherwise viable but nonculturable (VBNC) organisms present in patient samples [10–12]. Recent studies have found that sub-inhibitory antibiotic concentrations can trigger the shift of *P. aeruginosa* to a VBNC state; and these were associated with poor lung function and more frequent exacerbations in cystic fibrosis patients [13, 14]. VBNC organisms lose the ability to grow on routine media but remain metabolically and transcriptionally active allowing their detection through nonculture-based methods including reverse transcriptase quantitative PCR (RT-qPCR) [12]. Recently, molecular based assays have been developed to rapidly identify pathogens. Molecular detection may increase the sensitivity for bacterial detection from samples such as sputum. In a recent study in patients with bronchiectasis a multiplex platform called the BioFire Film Array Pneumonia Plus Panel increased the detection of bacterial pathogens at exacerbation in 120 patients with bronchiectasis from 68% detected by culture to 86% detected using PCR. This assay utilises a nested multiplex polymerase chain reaction (PCR) approach targeting nucleic acids (DNA and RNA) to detect nine viruses and 18 bacteria and seven antimicrobial genes [15].

European Respiratory Society guidelines recommend early eradication of *P. aeruginosa* with antibiotic therapy, emphasising the importance of detecting this bacteria early and accurately [16]. Reported success of eradication therapy in bronchiectasis is around 40%. This is lower than is reported in cystic fibrosis (60–90%), which may be attributed to later diagnosis and less frequent monitoring for new infections. *P. aeruginosa* detection changes clinical practice, with different antibiotic management at exacerbation, and a different algorithm for treatment involving inhaled antibiotics in patients with frequent exacerbations [17, 18]. Consequently, a test that could identify *P. aeruginosa* (in sputum) earlier in the disease course, and with higher sensitivity than culture (the current clinical standard of care), would be of high clinical value.

The BioFire Film Array Pneumonia Plus Panel is designed for DNA and RNA detection and may not be suitable for use in treatment monitoring or in patients receiving antibiotics, because it may not distinguish between viable and nonviable organisms [19–21]. An attractive target that has shown correlation with viability is ribosomal RNA (rRNA) [22, 23]. There is precedent for the use of rRNA for the detection and quantification of viable bacteria such as for the accurate detection and quantification of *Mycobacterium tuberculosis* in sputum [24, 25]. This assay has shown great promise for monitoring *M. tuberculosis* infections, with comparable performance to current gold standards for diagnosis and greater accuracy than culture for monitoring treatment response [24].

Here we describe the clinical utility of a newly developed *P. aeruginosa-*specific bacterial quantification assay (BQA), targeting rRNA for the detection of viable *P. aeruginosa* from clinical samples in bronchiectasis patients.

## Methods

### Patient cohorts

**FIGURE 1:**
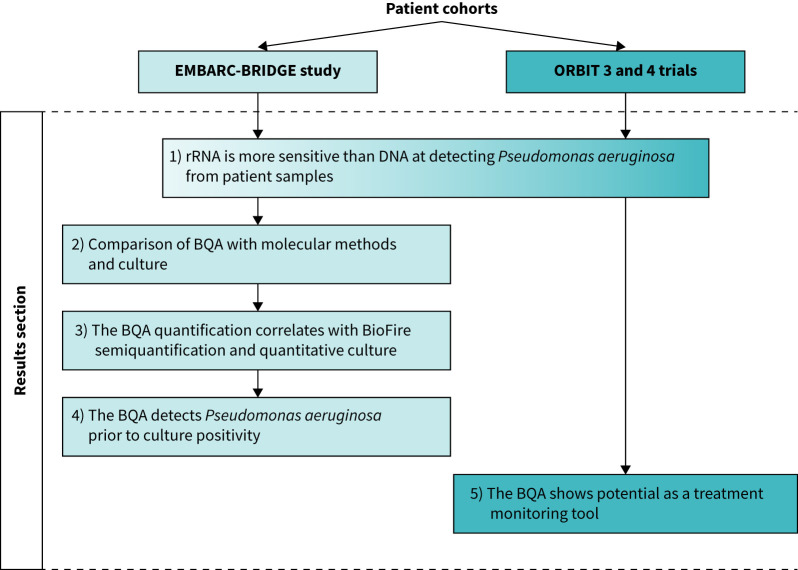
Illustration depicting the patient cohorts used for each result section. rRNA: ribosomal RNA; BQA: bacterial quantification assay.

Patient cohorts were used to address three clinical questions (summarised in figure 1): firstly, how detection of *P. aeruginosa* using the BQA compared to culture, and DNA-based molecular pathogen detection; secondly, based on higher sensitivity of molecular testing compared to culture, whether *P. aeruginosa* infection could be identified earlier in patients with bronchiectasis prior to their first positive culture; and finally, to evaluate whether the BQA could be used to monitor response to treatment using samples from patients enrolled in a randomised controlled trial of an inhaled antibiotic treatment to determine if the BQA could identify clearance of *P. aeruginosa* (referred to as eradication) and detect early relapse.

To answer these questions sputum samples were obtained from two bronchiectasis patient cohorts. For both cohorts, sputum was obtained from participants and stored at −80°C prior to processing.

The EMBARC-BRIDGE study is a multicentre prospective observational study embedded within the European Bronchiectasis Registry [4, 26, 27]. Patients enrolled into the EMBARC-BRIDGE study provided sputum samples at a baseline visit and used for molecular microbiology. Where sufficient sample was available, sputum from the same clinical visit was sent for routine clinical microbiology to compare culture results with the results of the molecular assays, and 16S rRNA gene sequencing was performed (as described below). The BRIDGE study is registered under ClinicalTrials.gov ID: NCT03791086.

The ORBIT 3 and 4 trials were double-blind, randomised, placebo-controlled trials of inhaled liposomal ciprofloxacin *versus* placebo in patients with chronic *P. aeruginosa* infection [28]. Sputum samples at monthly study visits were sent to the central lab for determination of bacterial load and detection of *P. aeruginosa* by quantitative culture. Samples were stored at −80°C and then transferred to the EMBARC bioresource for further analysis at the end of the trial. Specific samples were selected for analysis of treatment response by identifying patients who had achieved “eradication”: defined as three or more negative monthly sputum samples following treatment, and no subsequent positive sputum samples for *P. aeruginosa* by culture. “Relapse” was defined as a minimum of two consecutive monthly negative sputum samples followed by a positive sample by culture. The ORBIT clinical trials were registered under ClinicalTrials.gov IDs: NCT01515007and NCT02104245.

### Prototype assay analytical performance

The rRNA BQA prototype assay analytical performance was evaluated. Where available methods were adapted from relevant Clinical Laboratory Standards Institute (CLSI) standards. Full details are described in the supplementary methods.

### RNA extraction

Sputum RNA was extracted using the ZymoBIOMICS RNA Miniprep kit (Zymo Research; catalogue number (Cat. No.) R2001) according to the manufacturer's instructions with small modifications. Full details are described in the supplementary methods.

### Dual nucleic acid extraction

Sputum RNA and DNA were extracted simultaneously using the AllPrep DNA/RNA Mini kit (Qiagen; Cat. No. 80204) according to the manufacturer's instructions with small modifications. Full details are described in the supplementary methods.

### RT-qPCR

The clinical utility of the BQA was evaluated in collaboration between LifeArc and the University of Dundee.

One-step RT-qPCR was performed to quantify *P. aeruginosa* rRNA from patient sputum samples. The general reaction mix per reaction used 1× master mix, 1× reverse transcriptase enzyme, optimised concentration of primers and dual-labelled probe (IDT) with water making up the total reaction volume to 8 µL. The dual-labelled probe was tagged with a 5′ 6-FAM reporter dye and a 3′ nonfluorescent quencher. A DNA contamination control mastermix (RT negative) was also prepared, to ensure rRNA quantification was reliable. The RT negative reaction mix was identical to the general mix except for absence of reverse transcriptase enzyme.

RT-qPCR was performed using a QuantStudio 7 Flex Real-Time PCR machine (Applied BioSystems). 2 µL of a 1 in 10 dilution from each sample was used per reaction. Samples, positive control and water controls were tested in triplicate. Samples with a quantification of ≥4.57 copies per reaction were considered positive for *P. aeruginosa.* Cut-off rationale is explained in the supplementary methods.

Standard curves were performed as required, with 1 in 10 serial dilutions prepared from a known concentration positive control to obtain copies per reaction for each sample.

Owing to plans for assay commercialisation, further details including oligo sequences, reaction components and cycling conditions cannot be disclosed.

### Multiplex PCR (BioFire)

Respiratory pathogens from sputum samples were identified by a nested multiplex PCR, BioFire Film Array Pneumonia Plus Panel with the FilmArray 2.0 multiplex PCR system (Biomerieux) as previously described [29].

### 16S rRNA gene sequencing (16S)

Sputum DNA was extracted using the DNeasy PowerSoil Pro Kit (Cat. No. 47014). DNA was sequenced by synthetic full-length 16S rRNA gene LoopSeq [30]. Reads quality was checked using FASTQC and MultiQC in R 4.1.2 (2022-12-30). ASVs were generated through DADA2 pipeline with taxonomy assignment to species level utilising Silva v.138.1 database [31]. Taxa identified were reported as a positive result if present with >10 reads or with >0.1% relative abundance in a sample. Sequencing data have been submitted to European Nucleotide Archive (ENA) (Project accession number PRJEB88963).

### Statistical analysis

Sensitivity and specificity were calculated as previously reported [32]. 95% confidence intervals (CI) were calculated utilising the percentile method using bootstrapping with 500 iterations. Correlation analyses were performed using either Spearman's rank or Pearson's correlation coefficient as appropriate.

## Results

### Analytical performance of the BQA

The analytical performance of the BQA was confirmed (supplementary methods). The BQA has a wide linear interval of 42–4.95×10^7^ copies per reaction, with low limit of quantification (LOQ) and limit of detection (LOD) of ≥688 and 40 copies per reaction, respectively. The assay was highly reproducible, with covariance of <10% for intra-assay precision and repeatability between assay days (supplementary methods and table S1).

### rRNA is more sensitive than DNA at detecting *P. aeruginosa* from patient samples

To demonstrate the suitability of the BQA to identify *P. aeruginosa* in patient sputum, 20 samples from the ORBIT clinical trials with culture-confirmed *P. aeruginosa* infection (bacterial load range of 1.6–9 log CFU per g) were tested. 100% concordance was found with the culture positive samples and rRNA BQA results (20/20 were positive by BQA).

**FIGURE 2:**
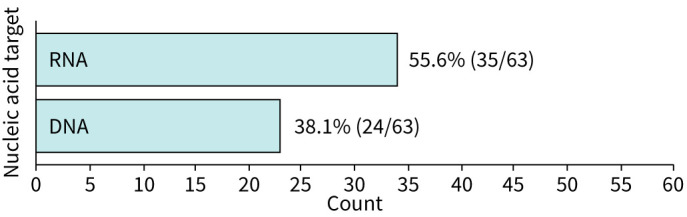
*Pseudomonas aeruginosa* bacterial quantification assay positive status count using ribsomal RNA and DNA as assay targets for samples from patients who were *P. aeruginosa* culture negative but went on to have *P. aeruginosa* detected *via* culture at a later date.

As most current molecular methods used for diagnostics target DNA, we compared the sensitivity of rRNA and DNA as targets for the BQA. Dual nucleic acid extractions were performed on samples with *P. aeruginosa* culture negative status from patients who went on to receive a positive culture status at a later date. Both DNA and rRNA were used as targets for the BQA, and their *P. aeruginosa* detection percentage was compared. Detection of *P. aeruginosa* was significantly higher when targeting rRNA compared to DNA (55.6% *versus* 38.1%; difference=17.5%, 95% CI: 0.3–34.6%; p=0.0495) (figure 2). rRNA quantification had a median of 1.04×10^3^ copies per reaction (range 5.67–4.73×10^6^) and DNA quantification had a median of 3.73×10^2^ copies per reaction (range 9.98–4.10×10^4^). This demonstrates the superior sensitivity of rRNA over DNA, and rRNA was used as the target for all further analysis. Full analysis details can be found in supplementary tables S2 and S3.

### Comparison of the BQA with molecular methods and culture

The performance of the BQA was compared with BioFire, culture and 16S rRNA gene sequencing. 111 samples obtained from 98 bronchiectasis patients as part of the EMBARC BRIDGE study were used in this investigation. 54 (55.1%) of patients were male, and mean±SD age was 64.8±14.1 years. The mean±SD BMI was 25.9±5.5 and FEV_1_ % predicted was 75.6±26.3%. 41 (41.8%) patients had had at least two exacerbations in the previous year. 38 (38.8%) patients had idiopathic bronchiectasis, 15 (15.3%) had post-infective bronchiectasis, 12 (12.2%) COPD, 9 (9.2%) primary ciliary dyskinesia, 7 (7.1%) asthma and 5 (5.1%) NTM. Other causes of bronchiectasis accounted for <four cases. All 111 samples were processed for both BioFire and the BQA; of these 57 had previous culture results and 62 had sequencing data available.

**FIGURE 3:**
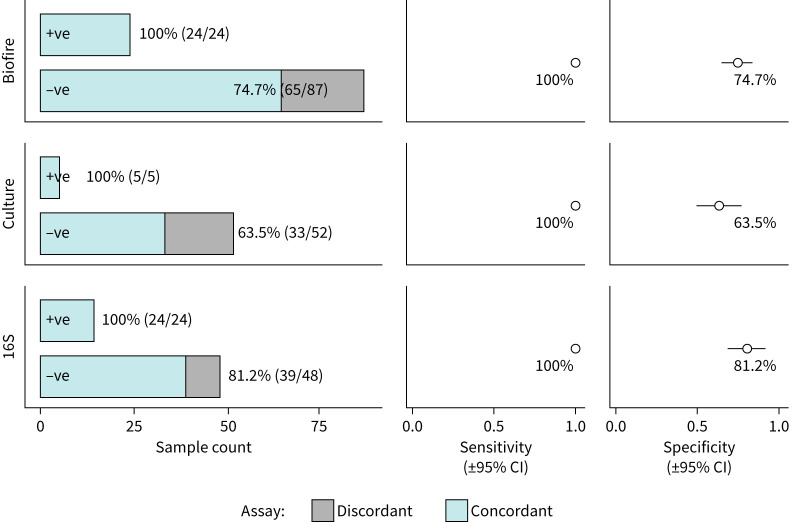
Sensitivity and specificity of the bacterial quantification assay when compared to the BioFire Pneumonia Plus Panel, Culture and 16S ribosomal RNA gene sequencing status.

Compared to BioFire, the BQA demonstrated a sensitivity of 100% (24 of 24; 95% CI: 100–100%) and a specificity of 74.7% (65 of 87; 95% CI: 64.4–83.4%), indicating that the BQA identified *P. aeruginosa* in 22 BioFire negative samples. Compared to culture, the BQA demonstrated a sensitivity of 100% (5 of 5; 95% CI: 100–100) and a specificity of 63.5% (33 of 52; 95% CI: 50.4–77.6), indicating that the BQA identified *P. aeruginosa* in 23 culture-negative samples. Compared to 16S rRNA gene sequencing, the BQA demonstrated a sensitivity of 100% (14 of 14; 95% CI: 100–100) and a specificity of 81.2% (39 of 48; 95% CI: 69.3–92.1), indicating that the BQA identified *P. aeruginosa* in nine sequencing negative samples (figure 3).

Neither culture nor DNA targeting methods can be considered a true gold standard, due to low sensitivity and inability to distinguish between viable and nonviable organisms respectively; therefore, it is difficult to establish whether the BQA is identifying clinically important *P. aeruginosa* which is missed by the other methods, or whether BQA identifies false positives.

### BQA quantification correlates with BioFire semi-quantification and quantitative culture

**FIGURE 4:**
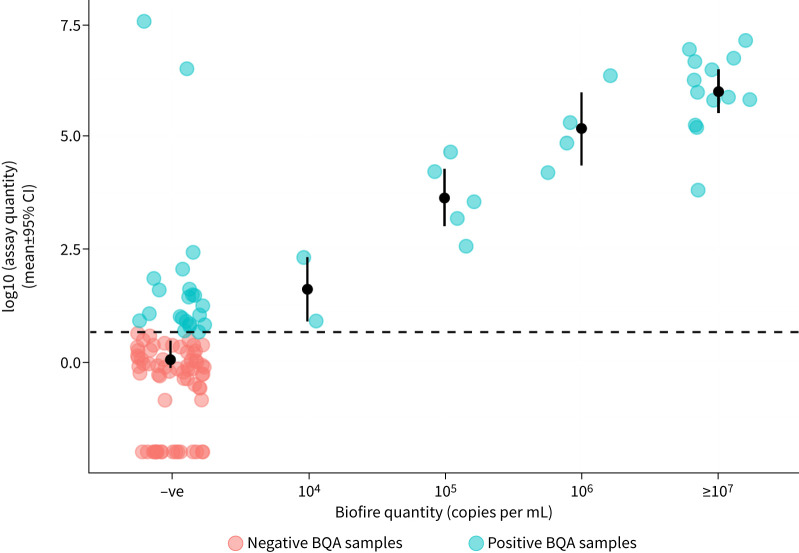
Comparison of BioFire and the bacterial quantification assay quantitative outputs (n=111). Each point represents a single sputum sample, and compares the results for both the bacterial quantification assay and BioFire. Dashed line represents the bacterial quantification assay cut-off (4.57 copies per reaction). BQA: bacterial quantification assay.

BioFire provides a semi-quantifiable result, so to investigate the relationship with the BQA quantification we performed a correlation analysis (figure 4). A strong positive correlation between BioFire and BQA quantification was observed (Spearman's ρ=0.68, 95% CI: 0.57–0.77, p<0.001).

**FIGURE 5:**
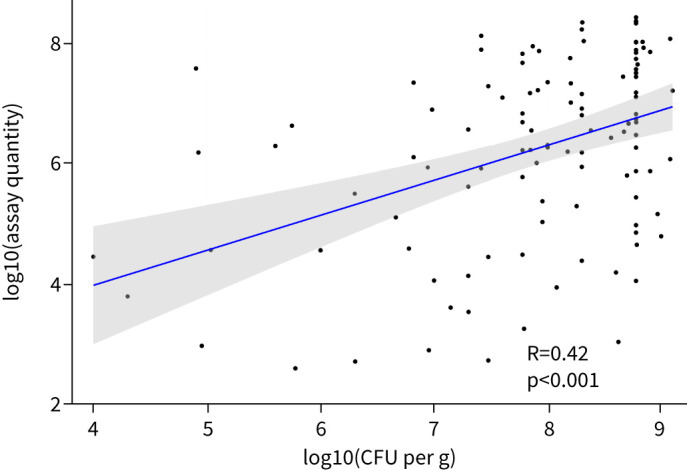
Comparison of quantitative culture (CFU per g) and the bacterial quantification assay quantitative outputs (n=113). Each point represents a single sputum sample, and the results for the bacterial quantification assay and quantitative culture. Blue line represents regression line, with 95% confidence interval shown in grey.

To determine the relationship of the BQA quantification with quantitative culture (CFU per g), 113 baseline samples from the ORBIT trials (*P. aeruginosa* culture positive) were used and a correlation analysis performed (figure 5). A moderate positive correlation was observed between quantitative culture and BQA quantification (Pearson's r=0.42, CI: 0.25–0.56, p<0.001).

### BQA detects *P. aeruginosa* prior to culture positivity

A benefit of RNA molecular-based methods for pathogen detection is their higher sensitivity and their ability to detect organisms that are VBNC. As such using a molecular-based method might allow earlier detection of pathogens than culture.

This analysis required identifying sputum samples that were culture negative for *P. aeruginosa* from patients who went on to develop a *P. aeruginosa* infection at a later date. 15 patients were identified who had a first or new isolation of *P. aeruginosa* during the BRIDGE study. Sputum samples which were negative for *P. aeruginosa* and obtained 8 to 484 days prior to primary *P. aeruginosa* detection by culture were then studied to identify if the BQA could identify *P. aeruginosa* earlier than culture.

**FIGURE 6:**
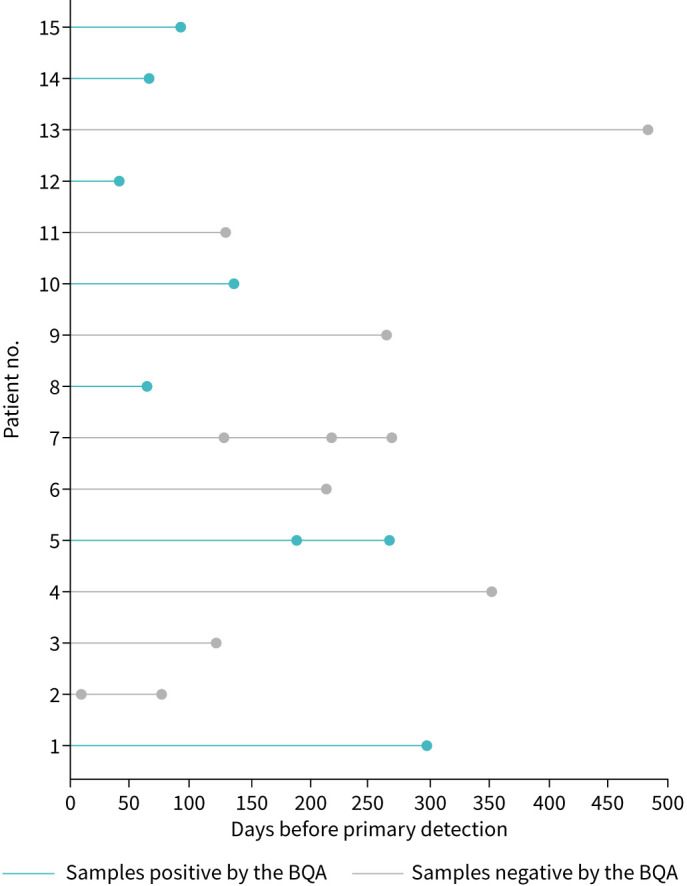
*Pseudomonas aeruginosa* bacterial quantification assay status of each sample (per patient) and the number of days before primary *P. aeruginosa* detection by culture. Patient number 2 has two overlapping points (Days 8 and 9). BQA: bacterial quantification assay.

8 of 20 (40.0%; exact 95% CI: 19.1–63.9%) samples from 7 out of 15 patients were positive by BQA for *P. aeruginosa* rRNA (figure 6). The *P. aeruginosa* rRNA concentration ranged from 5.47×10^1^ to 7.28×10^6^ copies per reaction, indicating a variable sample *P. aeruginosa* bacterial load. The mean number of days prior to culture detection was 138 (range 41–298). Full analysis details can be found in supplementary table S2.

These findings indicate the potential for the BQA to detect *P. aeruginosa* at an earlier timepoint than culture-based methods in a subset of patients. Owing to the small sample size and variability between patients, this analysis should be interpreted as preliminary, with larger studies required to validate findings.

### BQA shows potential as a treatment monitoring tool

To explore the potential of the BQA for infection treatment monitoring, 36 patients were selected from the ORBIT clinical trials based on *P. aeruginosa* infection status. Infection status was designated as eradication (n=10) or relapse (n=27, one patient had two relapses). Eradication was defined by three consecutive negative sputum cultures, and relapse by a *P. aeruginosa* positive sample following at least two consecutive negative samples. The BQA quantification was compared to quantitative culture (CFU per g) for concordance.

**FIGURE 7:**
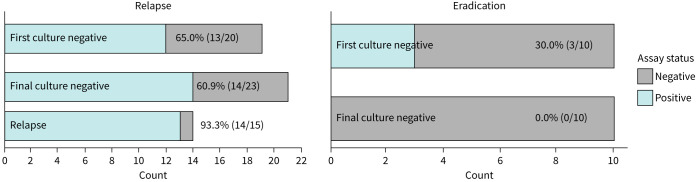
*Pseudomonas aeruginosa* bacterial quantification assay status for samples from patients who went on to a *P. aeruginosa* relapse or eradication with relation to their culture status.

For the relapse cohort two culture negative samples (first available culture negative and final available culture negative prior to relapse) and the positive sample indicating relapse were selected (where available). 13 of 20 (65.0%; exact 95% CI: 40.8–84.6%) “first culture negative” samples were positive by BQA (figure 7). The trend continued with the “final available culture negative” samples with 14 of 23 (60.9%; exact 95% CI: 38.5–80.3%) being positive (figure 7). Lastly, 14 of 15 (93.3%; exact 95% CI: 68.1–99.8%) relapse samples were positive, with the one negative sample having a 1.6 log CFU per g by culture (figure 7).

For the eradication cohort two culture negative samples (first available culture negative and further culture negative prior to end of the clinical trial) were selected. 3 of 10 (30%; exact 95% CI : 6.7–65.2%) “first culture negative” samples were positive, with no positive samples at a later timepoint (figure 7). Full details of the analysis can be found in supplementary table S3.

## Discussion

We have described the clinical utility of a newly developed BQA for the detection of viable *P. aeruginosa* from bronchiectasis patient samples. The main goal was to determine the suitability of the BQA in a clinical setting. To do so we compared the assay performance with the current clinical standard of care (culture), and two additional methods (16S rRNA gene sequencing and the molecular based assay BioFire Film Array Pneumonia Plus Panel). The BQA showed high sensitivity when compared to all three methods (100%), and also identified a high proportion of additional positive samples which were not identified by these other methods. This could indicate either superiority of the BQA, or a lack of specificity (“false positives”). A major challenge in the development of a novel assay like this is that there is no true gold standard, since culture is known to have poor sensitivity. As such, it is not certain that all of the additional positive *P. aeruginosa* detections with the BQA are clinically significant. We therefore took advantage of the availability of unique well-characterised patient cohorts to explore scenarios where the BQA could be used clinically. Several findings from testing these samples indicate that positive detection of *P. aeruginosa* using BQA when other assays tested negative is due to increased sensitivity of this assay and not decreased specificity. First, other identification that there were no “false positives” using the BQA at late timepoints after successful eradication makes it less likely that the BQA has lower specificity than the other assays. Second, the superior sensitivity compared to the other assays in patients who subsequently relapsed (tested positive by culture) supports the conclusion that the BQA is more sensitive but remains highly specific. An additional benefit of the BQA is quantification, which can be useful for assessing clinical significance and monitoring treatment response. The BQA quantification showed a strong positive relationship with BioFire quantification.

Alongside higher sensitivity compared to culture, it has been shown that molecular methods can detect VBNC. It is believed that initial infection with *P. aeruginosa* is likely to be preceded by either prior infections with *P. aeruginosa* that are cleared, or low-level *P. aeruginosa* infection that may evade detection until it comes to dominate the microbiome [33]. Therefore, there is theoretically an opportunity for molecular methods to identify *P. aeruginosa* infection prior to the first culture positive. Here we showed that the BQA detected *P. aeruginosa* up to 298 days prior to primary detection by culture. The data from the EMBARC cohort are limited by the variable timepoints at which the prior sputum samples were obtained as part of routine clinical practice. Therefore, the possibility of a new infection developing between BQA positive and culture positive timepoints cannot be fully excluded. Nevertheless, the ability to detect *P. aeruginosa* infection earlier was confirmed by the “relapse” analysis in the ORBIT trials where monthly samples prior to a positive sample showed that culture negative samples were positive earlier with the BQA. It is not possible to definitively ascertain whether the BQA positivity is due to higher sensitivity compared to culture or due to detection of VBNC; however, as the range of copies per reaction was 5.67–4.73×10^6^, it would suggest that positive results may be due a combination of these. In a clinical setting, earlier detection may have allowed treatment to begin sooner. It is believed that eradication treatment, as recommended by European Respiratory Society (ERS) guidelines, is likely to be more effective if started closer to the date of first infection [34]. Although both nucleic acids were positive during culture negative states using the BQA, rRNA was shown to be more sensitive than DNA in detecting a higher number of positive samples, highlighting a potential benefit of using rRNA as the target for this BQA assay.

The BQA detected *P. aeruginosa* in culture negative samples prior to infection relapse. This is unlikely to be due to rRNA survival following cell death, as for three of the patients the second culture negative sample had a higher copy number than the first with a time gap of 4 to 24 weeks between samples. Furthermore, no further culture negative samples from patients who went on to *P. aeruginosa* eradication were BQA positive, with a time gap of 4 to 40 weeks between samples. This is an initial indication of the BQA's ability as a treatment monitoring tool, but further validation is required due to small patient numbers. Patients with three consecutive negative samples were chosen as the most likely to be genuine eradications. The lack of positive results in these samples supports that genuine false positive results with the BQA are unlikely.

Apart from utility in a clinical setting, another potential BQA application is clinical trials where monitoring the efficacy of treatments is crucial. Current methods to monitor bacterial infection for clinical trials are culture based; this is labour intensive and requires processing of samples in a timely manner that either requires the rapid shipment of samples to a central facility, which is costly, or processing at different centres, which may lead to inconsistencies [28]. Importantly, although culture is processed during the trial, assessment of treatment effects of bacterial infection is performed retrospectively at the end of the study. In comparison, if the BQA is shown as a suitable alternative, samples could be appropriately stored and processed at the end of the trial with a substantial cost saving. This should be evaluated in future studies. Although these results are encouraging for a role of molecular testing in future trials or clinical practice, additional questions and limitations of molecular tests will need to be considered. Further studies are needed to understand whether treatment of BQA positive, but culture negative, patients results in clinical benefit such as more effective eradication. BQA does not provide direct susceptibility data, in contrast to culture. While the clinical relevance of susceptibility data for *P. aeruginosa* is often questioned, for clinical trials, *e.g.* of inhaled antibiotics, it is often important to understand whether resistance arises, and therefore BQA may need to be combined with culture.

We conducted initial investigation and assessment of the assay in small subsets of patients with specific characteristics, but larger scale clinical testing of the assay is now required in unselected patient groups.

In conclusion, we have developed and evaluated a novel BQA for the detection of viable *P. aeruginosa* from bronchiectasis patient samples. The BQA has shown comparability with current methods used in a clinical setting, with the potential to detect *P. aeruginosa* prior to primary culture identification and to detect *P. aeruginosa* in culture negative samples prior to an infection relapse. The BQA results demonstrate potential for clinical application in early diagnosis and treatment monitoring.

## Supplementary material

**Please note:** supplementary material is not edited by the Editorial Office, and is uploaded as it has been supplied by the author. Supplementary material 01389-2025.SUPPLEMENT
