# Supplementary material for: Assessment of a bacterial quantification assay to detect and monitor Pseudomonas aeruginosa infection in patients with bronchiectasis
Source: ERJ Open Res. 2026 Aug 3;12(4):01389-2025. doi: 10.1183/23120541.01389-2025 (PMC13430582; doi:10.1183/23120541.01389-2025)
Supplement: Supplementary file 1 [file 01389-2025.SUPPLEMENT.pdf]

Supplement

Contents

Supplementary methods 1

Supplementary Data - Results 3

Supplementary references 8

Supplementary methods

RNA extraction from sputum

In brief, 0.1g of sputum was homogenised in ZR bashing bead lysis tubes with DNA/RNA shield (1000µl of 1x or 650 µl of 2x concentration) for 40 seconds with a 15 second pause at 6200rpm using a Precellys instrument. 2 volumes of RNA lysis buffer were added to the supernatant, the full sample was then processed according to manufacturers' instructions with DNAse I treatment.

For RNA extractions where *Staphylococcus aureus* was suspected, a lysostaphin step was performed prior to mechanical lysis. The addition of lysostaphin was shown to not impact *P. aeruginosa* yield (unpublished data).

Dual nucleic acid extraction

In brief, 0.1g of sputum was homogenised in ZR bashing bead lysis tubes with 600µl of Buffer RLT plus with β-Mercaptoethanol for 40 seconds with a 15 second pause at 6200rpm using a Precellys instrument. The full sample was then processed according to manufacturers' instructions. The RNA sample was DNAse I treated with RNA cleanup using the RNeasy Mini kit following manufacturers' instructions (Qiagen Cat. No. 74104).

RT-qPCR background amplification cut-off

To determine *P. aeruginosa* positive cut-off, the average quantification for Cq 35 of 14 plates was used. This provided us with 4.57 copies/reaction as our cut-off. Cq 35 cut-off reliably differentiated between true and background amplification during assay development activities. Cq 35 had been determined as a cut-off for background amplification during assay development (data not shown).

Bacterial culture

P. aeruginosa (NCTC 12903) cultures were grown at 37°C in LB Miller broth, with an initial overnight culture followed by a subculture in fresh LB Miller broth for 4 hours.

RNA extraction from bacterial culture

RNA was extracted as described above with small modifications. 1.5ml of pelleted *P. aeruginosa* culture resuspended in 350 µl residual media/PBS was homogenised with 700 µl of DNA/RNA shield in ZR bashing bead lysis tubes for 40 seconds at 6000rpm using a Precellys instrument. The remainder of the extraction was performed as previously described.

Prototype assay analytical performance

The prototype assay analytical performance was evaluated. Template consisted of *P. aeruginosa* total RNA extracted from culture. Quantities for RNA were determined using standard curves generated from a 10-fold dilution series of a positive control at known concentrations (100 – $1x10^7$ copies/reaction). Key performance characteristics of intra-assay precision, repeatability, limit of detection (LOD), limit of quantification (LOQ), and linearity were assessed. Where available methods were adapted from relevant Clinical Laboratory Standards Institute (CLSI) standards.

Intra-assay precision was assessed in single RT-qPCR runs performed by operators paired with either a QuantStudio 6 Real-Time PCR System (QS6) or a Quant Studio 7 Flex Real-Time PCR System (QS7). Total RNA with rRNA concentrations of approximately $1x10^6$

copies/reaction (High), $1x10^4$copies/reaction (Medium), and $1x10^3$ copies/reaction (Low) were tested up to 42 times each. The percentage coefficient of variation (%CV) in reported quantities was used as the measure of intra-assay precision.

Methods of assessing repeatability were adapted from CLSI EP05A-A3 (1). The assessment followed a 20 x 4 x 1 manner, with testing across 20 days, 1 run per day, with 4 replicates per run of total RNA at High, Medium, and Low rRNA concentrations.

The LOD and LOQ were assessed across a 2-fold dilution series of total RNA with rRNA concentrations of 21- $2.2x10^4$ copies/reaction. Data was collected from 90 replicates per concentration. Data analysis was performed using the method reported previously (2), which are similar to the probit approach outlined in CLIS EP17-A2 (3). The LOD was defined as the concentration predicted to yield 95% positive reactions. The LOQ was defined as the lowest assessed concentration with 100% positive reactions and %CV ≤35.

The linear interval of the assay was determined with methods based on the relative concentration approaches outlined in CLSI EP06 (4). During the analysis, the expected concentrations of each dilution were determined relative to the reported concentration of the most concentrated dilution. The relationship between reported and expected concentrations were modelled with a weighted least squares (WLS) regression. Predicted concentrations were calculated using this WLS model and the deviation between reported and predicted concentrations determined. The linear internal was considered the concentration range with absolute deviations ≤ 30%.

Supplementary results

Prototype Analytical Performance

The *P. aeruginosa* bacterial quantification assay (BQA) analytical performance were assessed including intra-assay precision, repeatability, LOD, LOQ and linearity (Table S1).

Table S1: Summary of prototype analytical performance assessment

**Characteristic**	**Result**
Intra-assay precision (%CV)	< 10%
Repeatability (%CV)	< 10%
Limit of detection	40 c/rx
Limit of quantification	688 c/rx
Linearity	Linear interval: 42 – 4.95×10^7 c/rx

c/rx: copies/reaction

Table S2: Detailed early detection analysis, including patient, status and quantification by rRNA and DNA

Patient	Status	PA CFU	rRNA Cq (mean)	DNA Cq (mean)	rRNA quantification (mean copies/reactions)	DNA quantification (mean copies/reactions)
1	298 days before culture positive	Neg	27.96	Neg	8.66E+02	Neg
2	8 days before culture positive	Neg	Neg	Neg	Neg	Neg
2	9 days before culture positive	Neg	Neg	Neg	Neg	Neg
2	76 days before culture positive	Neg	Neg	Neg	Neg	Neg
3	124 days before culture positive	Neg	Neg	Neg	Neg	Neg
4	358 days before culture positive	Neg	Neg	Neg	Neg	Neg
5	266 days before culture positive	Neg	28.33	35.13	6.75E+02	9.98E+00
5	190 days before culture positive	Neg	Neg	Neg	Neg	Neg
6	216 days before culture positive	Neg	Neg	Neg	Neg	Neg
7	218 days before culture positive	Neg	Neg	Neg	Neg	Neg
7	127 days before culture positive	Neg	32.12	Neg	6.24E+01	Neg
7	268 days before culture positive	Neg	Neg	Neg	Neg	Neg
8	63 days before culture positive	Neg	32.06	Neg	5.47E+01	neg
9	266 days before culture positive	Neg	Neg	Neg	Neg	Neg
10	137 days before culture positive	Neg	17.94	26.26	7.28E+05	2.70E+03
11	129 days before culture positive	Neg	Neg	Neg	Neg	Neg
12	41 days before culture positive	Neg	27.65	33.51	1.07E+03	2.29E+01
13	484 days before culture positive	Neg	Neg	Neg	Neg	Neg
14	67 days before culture positive	Neg	23.56	28.11	1.66E+04	7.83E+02
15	93 days before culture positive	Neg	22.95	25.86	2.52E+04	3.53E+03

Table S3: Detailed relapse and eradication analysis, including study question, patient, visit, status and quantification by rRNA and DNA

Study question (Patient)	Visit	Status	Log PA CFU	RNA Cq (mean)	DNA Cq (mean)	RNA quantification (mean copies/reactions)	DNA quantification (mean copies/reactions)
Relapse 1	9	1st culture negative	Neg	Neg	Neg	Neg	Neg
	10	Culture negative before relapse	Neg	Neg	Neg	Neg	Neg
	11	Relapse	1.60	Neg	Neg	Neg	Neg
Relapse 2	6	1st culture negative	Neg	21.79	29.16	5.60E+04	3.94E+02
	7	Culture negative before relapse	Neg	24.68	32.04	8.13E+03	5.83E+01
	9	1 month after relapse	6.60	22.74	30.03	2.89E+04	2.21E+02
Relapse 3	9	1st culture negative	Neg	34.95	Neg	5.67E+00	Neg
	10	Culture negative 2 months before relapse	Neg	30.74	Neg	9.60E+01	Neg
	14	2 month after relapse	8.30	13.75	20.90	9.59E+06	7.50E+04
Relapse 4	7	1st culture negative	Neg	18.78	27.33	4.09E+05	1.34E+03
	8	Culture negative 2 months before relapse	Neg	27.71	22.88	1.04E+03	2.64E+04
	10	Relapse	7.50	30.14	21.05	1.16E+06	9.00E+04
Relapse 5	2	1st culture negative	Neg	26.93	30.10	1.75E+03	2.10E+02
	7	Culture negative before relapse	Neg	30.11	Neg	2.09E+02	Neg
	8	Relapse	3.90	29.54	Neg	3.55E+02	Neg
Relapse 6	2	Culture negative 14d after 1st culture negative	Neg	Neg	Neg	Neg	Neg
	7	Culture negative 3 months before relapse	Neg	Neg	Neg	Neg	Neg
	10	Relapse	8.70	18.06	20.62	7.43E+05	1.21E+05
Relapse 7	10	Culture negative 1m after 1st culture negative	Neg	Neg	Neg	Neg	Neg
	11	Culture negative 2 months before relapse	Neg	31.90	Neg	6.38E+01	Neg

	14	1 month after relapse	8.60	15.89	24.07	2.83E+06	1.19E+04
Relapse 8	5	1st culture negative	Neg	Neg	Neg	Neg	Neg
	7	Culture negative before relapse	Neg	Neg	Neg	Neg	Neg
	8	Relapse	2.80	24.64	26.48	6.54E+03	1.91E+03
Relapse 9	9	1st culture negative	Neg	30.98	33.51	9.39E+01	1.81E+01
	12	Culture negative before relapse	Neg	32.51	33.94	3.41E+01	1.30E+01
	14	1 month after relapse	1.60	21.40	24.76	5.74E+04	6.07E+03
Relapse 10	11	1st culture negative	Neg	30.94	31.58	9.63E+01	6.33E+01
	13	Culture negative before relapse	Neg	Neg	Neg	Neg	Neg
	14	Relapse	6.30	27.89	Neg	7.44E+02	Neg
Relapse 11	9	1st culture negative	Neg	15.11	22.60	3.90E+06	2.58E+04
	12	Culture negative before relapse	Neg	24.92	29.84	5.43E+03	2.03E+02
Relapse 12	11	1st culture negative	Neg	19.44	29.01	2.14E+05	3.51E+02
	12	Culture negative 2m before relapse	Neg	14.82	21.90	4.73E+06	4.10E+04
Relapse 13	6	1st culture negative	Neg	Neg	Neg	Neg	Neg
	8	Culture negative before relapse	Neg	33.08	Neg	2.37E+01	Neg
Relapse 14	2	1st culture negative	Neg	Neg	Neg	Neg	Neg
Relapse 15	11	1st culture negative	Neg	15.89	23.78	2.46E+06	1.20E+04
	12	Culture negative 2 months before relapse	Neg	33.54	Neg	1.70E+01	Neg
Relapse 16	9	Culture negative before relapse	Neg	32.35	32.35	3.79E+01	3.74E+01
	10	Relapse	8.60	15.49	24.19	3.19E+06	9.09E+03
Relapse 17	2	Culture negative 2 months before relapse	Neg	Neg	Neg	Neg	Neg
Relapse 18	2	1st culture negative	Neg	22.09	26.25	3.75E+04	2.27E+03
Relapse 19	2	1st culture negative	Neg	31.48	34.01	6.71E+01	1.27E+01
Relapse 20	8	1st culture negative	Neg	27.55	Neg	9.47E+02	Neg

	9	Culture negative 2 months before relapse	Neg	24.79	33.35	6.07E+03	1.93E+01
	11	Relapse	7.78	15.99	25.45	2.27E+06	3.90E+03
Relapse 21	11	1st culture negative	Neg	Neg	Neg	Neg	Neg
	12	Culture negative before relapse	Neg	Neg	Neg	Neg	Neg
Relapse 22	11	1st culture negative	Neg	20.86	27.05	8.58E+04	1.32E+03
Relapse 23	7	Culture negative before relapse	Neg	Neg	Neg	Neg	Neg
	8	Relapse	6.78	19.54	27.46	2.09E+05	1.01E+03
Relapse 24	8	Culture negative 2 months before relapse	Neg	Neg	Neg	Neg	Neg
Relapse 25	11	Culture negative before relapse	Neg	Neg	Neg	Neg	Neg
	12	Relapse	2.90	32.10	Neg	4.43E+01	Neg
Relapse 26	12	Culture negative 2 months before relapse	Neg	20.11	26.11	1.42E+05	2.50E+03
	14	Relapse	7.00	21.57	28.25	5.34E+04	5.93E+02
Relapse 27	7	Culture negative before relapse	Neg	30.30	Neg	1.49E+02	Neg
Eradication 1	7	2m after 1st culture negative	Neg	33.52	Neg	1.62E+01	Neg
	11	culture negative	Neg	Neg	Neg	Neg	Neg
Eradication 2	11	2m after 1st culture negative	Neg	Neg	Neg	Neg	Neg
	12	culture negative	Neg	Neg	Neg	Neg	Neg
Eradication 3	8	1st culture negative	Neg	Neg	Neg	Neg	Neg
	11	culture negative	Neg	Neg	Neg	Neg	Neg
Eradication 4	2	1st culture negative	Neg	Neg	Neg	Neg	Neg
	12	culture negative	Neg	Neg	Neg	Neg	Neg
Eradication 5	9	1st culture negative	Neg	20.48	23.19	1.02E+05	1.66E+04
	12	culture negative	Neg	Neg	Neg	Neg	Neg
Eradication 6	2	1st culture negative	Neg	31.21	Neg	7.73E+01	Neg
	10	culture negative	Neg	Neg	Neg	Neg	Neg

Eradication 7	12	1st culture negative	Neg	Neg	Neg	Neg	Neg
	15	culture negative	Neg	Neg	Neg	Neg	Neg
Eradication 8	2	1st culture negative	Neg	Neg	Neg	Neg	Neg
	11	culture negative	Neg	Neg	Neg	Neg	Neg
Eradication 9	2	1st culture negative	Neg	Neg	Neg	Neg	Neg
	8	culture negative	Neg	Neg	Neg	Neg	Neg
Eradication 10	2	1m after 1st culture negative	Neg	Neg	Neg	Neg	Neg
	9	culture negative	Neg	Neg	Neg	Neg	Neg

Supplementary references

1. CLSI. *Evaluation of Precision of Quantitative Measurement Procedures; Approved Guidelines – Third Edition*. CLSI document EP05-A3. Wayne, PA. Clinical Laboratory Standards Institute; 2012

2. Forootan A, Sjöback R, Björkman J, Sjögreen B, Linz L, Kubista M. Methods to determine limit of detection and limit of quantification in quantitative real-time PCR (qPCR). Biomol Detect Quantif. 2017 Apr 29;12:1-6. doi: 10.1016/j.bdq.2017.04.001. PMID: 28702366; PMCID: PMC5496743

3. CLSI. *Evaluation of Detection Capability for Clinical Laboratory Measurement Procedures; Approved Guideline – Second Edition*. CLSI document EP17-A2. Wayne, PA. Clinical Laboratory Standards Institute; 2012

4. CLSI. *Evaluation of Linearity of Quantitative Measurement Procedures.* 2nd ed. CLSI guideline EP06. Clinical Laboratory Standards Institute; 2020
